# Disorder by design: A data-driven approach to amorphous semiconductors without total-energy functionals

**DOI:** 10.1038/s41598-020-64327-3

**Published:** 2020-05-08

**Authors:** Dil K. Limbu, Stephen R. Elliott, Raymond Atta-Fynn, Parthapratim Biswas

**Affiliations:** 1 Department of Physics and Astronomy, The University of Southern Mississippi, Hattiesburg, Mississippi 39406 USA; 2 Department of Chemistry, University of Cambridge, Cambridge, CB2 1EW United Kingdom; 3 Department of Physics, The University of Texas at Arlington, Texas, 76019 USA

**Keywords:** Atomistic models, Computational methods, Electronic structure, Structure of solids and liquids

## Abstract

X-ray diffraction, Amorphous silicon, Multi-objective optimization, Monte Carlo methods. This paper addresses a difficult inverse problem that involves the reconstruction of a three-dimensional model of tetrahedral amorphous semiconductors via inversion of diffraction data. By posing the material-structure determination as a multiobjective optimization program, it has been shown that the problem can be solved accurately using a few structural constraints, but no total-energy functionals/forces, which describe the local chemistry of amorphous networks. The approach yields highly realistic models of amorphous silicon, with no or only a few coordination defects (≤1%), a narrow bond-angle distribution of width 9–11.5°, and an electronic gap of 0.8–1.4 eV. These data-driven information-based models have been found to produce electronic and vibrational properties of *a*-Si that match accurately with experimental data and rival that of the Wooten-Winer-Weaire models. The study confirms the effectiveness of a multiobjective optimization approach to the structural determination of complex materials, and resolves a long-standing dispute concerning the uniqueness of a model of tetrahedral amorphous semiconductors obtained via inversion of diffraction data.

## Introduction

Experimental information from scattering measurements, such as X-ray, electron and neutron diffraction, play an important role in the structural determination of ordered and disordered materials^1,2^. Diffraction experiments typically provide scattering intensities from the constituent atoms in the wavevector space, which are related to the two-body correlation function between atoms in real space via the Fourier transform of the structure factor. For amorphous solids, the reconstruction of the correct three-dimensional real-space structure from scattering data is an archetypal example of inverse problems in computational modeling of materials. Since the lack of information from higher-order atomic-correlation functions cannot be remedied by any amount of computational/mathematical trickeries, it is absolutely necessary to supplement experimental data by additional information to determine a unique structural model of a material. The term unique here refers to the unique macroscopic properties of a noncrystalline material obtained from the configurational averaging of an ensemble of similar structural solutions or atomistic models from simulations. While these solutions may differ from each other microscopically, due to configuration fluctuations, a minimal amount of structural information is necessary to reconstruct these microscopic solutions via an inverse approach. In a laboratory-grown bulk sample of noncrystalline materials, all possible disordered configurations are simultaneously present and their effects are reflected in experimental data for physical observables. Among earlier approaches to address the problem, the reverse Monte Carlo (RMC) method^3^ is an elegant method that relies on the generation of Markov chains or random walks to optimize a suitable cost function in the state-vector or configurational space. While a variant of the RMC method was used by Strong and Kaplow^4,5^ as early as in the 1960s to predict the structure of crystalline B_2_O_3_^4^ and vitreous selenium^5^, by refining X-ray diffraction data via random walks, it was McGreevy and co-workers^3,6,7^ who first applied the method systematically to model the structure of disordered solids^3^. Since then, the method has been widely used to study a variety of complex disordered systems, including liquids, glasses, disordered alloys, and proteins^7^.

Despite significant efforts over the past decades, direct application of the RMC method to determine reliable structural solutions of disordered materials from their diffraction data has remained a challenging problem to date. The problem is particularly acute for amorphous tetrahedral semiconductors, such as amorphous silicon (*a*-Si) and germanium (*a*-Ge). Although a number of RMC or RMC-derived studies^3,6–14^ on *a*-Si/*a*-Ge have been reported in the past, none of these could demonstrate the presence of a gap in the electronic spectrum and a low concentration of coordination defects (≤1%), as observed in optical measurements^15,16^ and electron spin resonance (ESR)^17^ experiments, respectively, for these materials. Thus, the problem of the structural determination of tetrahedral amorphous semiconductors from diffraction data *without* using a total-energy functional remains unsolved up until now. Since an ensemble of three-dimensional structures can lead to an identical two-body correlation function, additional information is required to uniquely determine the correct structure of a material by suitably reducing the volume of the solution space. While this reduction is generally achieved by imposing structural constraints during RMC simulations, the hierarchy and conflictive nature of the constraints render the resulting optimization problem very difficult, leading to poor structural solutions. To overcome this problem, a new breed of hybrid approaches have been developed in recent years^18–22^, which can successfully address the uniqueness problem by simultaneously employing experimental data and a total-energy functional. However, these hybrid approaches crucially rely on the availability of suitable total-energy functionals and, for many multinary systems, it is often difficult to guide the hybrid solutions without resort to the use of expensive quantum-mechanical force fields. This considerably increases the computational complexity of the problem, which limits the applicability of *ab initio*-based hybrid approaches in addressing large disordered systems. This necessitates the development of purely data-driven information-based approaches to material simulations, which rely on experimental data and auxiliary structural information.

The principal aim of this paper is to demonstrate conclusively that an accurate structural solution for tetrahedral amorphous semiconductors can be obtained *without* the need for a total-energy functional or atomic forces but using diffraction data only, assisted by a few structural constraints. By developing an efficient constraint optimization scheme, within the framework of Monte Carlo methods, which scales linearly with the system size, we show that tetrahedral models of *a*-Si can be constructed by the judicious use of local structural/chemical constraints and diffraction data. More importantly, we demonstrate that the data-driven information-based models are energetically stable in the sense that the models correspond to a stable local minimum of a quantum-mechanical total-energy functional and that the models produce a clean gap in the electronic spectrum. Furthermore, we show that the resulting models exhibit structural, electronic, and vibrational properties of *a*-Si that match excellently with the corresponding experimental data from X-ray, Raman spectroscopy, and inelastic neutron-diffraction measurements, as well as those obtained from using conventional simulation techniques, based on classical and quantum-mechanical force fields. Finally, we discuss an extension of the scheme with a few examples to incorporate microstructural properties of realistic samples of *a*-Si and *a*-Ge as observed in experiments.

## Results

In discussing our results, we first demonstrate the superior structural quality of the new models over existing RMC or RMC-like models. This is followed by an extensive comparison of the results from our models with those from conventional Monte Carlo and molecular-dynamics simulations, using classical, quantum-mechanical, and machine-learning-based potentials. We have placed great emphasis on the electronic structure of *a*-Si, as it is the electronic properties that are most difficult to produce accurately, not only in RMC/RMC-like methods but also in MD and MC simulations, and are often not discussed in the literature with sufficient detail. We then proceed to compare our results with hybrid models that employ both total-energy functionals and experimental data. This is followed by new ideas that serve as natural corollaries of the current approach in an effort to address microstructural properties of experimentally obtained *a*-Si samples, which cannot be described using conventional continuous random network (CRN) models of *a*-Si. Since the multiobjective constraint optimization problem in the present study was handled by using constraint Monte Carlo (CMC) methods, we shall refer to our models as CMC19 hereafter.

### Comparison with earlier RMC-derived models

To examine the accuracy of structural quantities and the novelty of the CMC19 approach, we begin by listing the characteristic properties of the CMC19 model in Table 1, along with those obtained from various RMC^9,10^ and RMC-derived^13,14,23^ approaches. A salient feature of all these methods is that none of the methods uses any total-energy functionals during structural formation but only experimental data in conjunction with a few constraints. The results presented in Table 1 clearly indicate that the CMC19 models outperform all other models, as far as the values of Δ*θ*, *c*_4_ and *E*_*g*_ are concerned. Although the lack of precise information on the bond-angle distribution from INVERT and SOAP models, in particular the root-mean-square (RMS) bond-angle width, Δ*θ*, makes it difficult to compare our results on a quantitative footing, it is evident that the INVERT and SOAP models produce too many defects (about 5–8%) to open a gap in the respective electronic spectrum. It may be noted that the structural and electronic quality of a model *a*-Si network is primarily determined by – apart from the structure factor or its real-space counterpart – a trinity of three quantities: (a) the width of the bond-angle distribution (Δ*θ*); (b) the concentration of 4-fold-coordinated atoms (*c*_4_) in a tetrahedral environment^24^; and (c) the value of the electronic gap (*E*_*g*_). It is therefore crucial for a realistic model of *a*-Si to exhibit a small value of Δ*θ* (of about 9–12°) and a large value of *c*_4_ (typically ≥ 98%). While an extension of the INVERT approach, EX-INVERT^23^, is reported to have produced highly 4-fold-coordinated networks with *c*_4_ values of up to 97%, the authors provided no data on the width of the bond-angle distribution and the statistics of atomic coordination in ref. ^23^. Given the multiobjective nature of the problem, it is possible to construct a network with a low concentration of coordination defects at the expense of a high value of Δ*θ*, and vice versa. However, such networks may not represent a stable physical solution upon total-energy relaxation. Thus, a structural model from a data-driven information-based approach must satisfy the aforementioned criteria simultaneously in order to be compliant with experimental data from Raman and optical measurements, and electron spin resonance experiments. By contrast, the CMC19 models obtained in the present study clearly and unambiguously satisfied all these criteria. It is remarkable that the 216-atom CMC19 model produces a 100% 4-fold-coordinated network with an RMS width of bond angles of about 10.95° and an electronic gap of magnitude 1.18 eV. We emphasize that this is the first ever data-driven information-based model that produces no defects and a pristine electronic gap, without using a total-energy functional during structural formation. This constitutes one of the major outcomes of the present study. In the following section, we shall demonstrate that even the unrelaxed CMC19 models of *a*-Si are capable of producing a gap in the electronic spectrum and that the structural properties of the models are energetically stable, i.e., the models are almost independent of *ab initio* total-energy relaxations.

**Table 1:** Comparison of results from various information-based approaches. *N*, 〈*θ*〉, Δ*θ*, *c*_4_ and *E*_*g*_ indicate the size of the system, the average bond angle, the RMS width of bond angles, the percentage of 4-fold-coordinated atoms and the value of the electronic gap in electron-volt, respectively.

Model	N	〈*θ*〉	Δ*θ*	*c*_4_	*E*_*g*_ (eV)
CMC19	216	109.08	10.95	100	1.18
CMC19	512	109.14	10.61	99.22	1.09
SOAP^§^	512	NA	NA	95	None
EX-INVERT^†^	216	NA	NA	94-97	NA
INVERT^‡^	512	NA	NA	92^§^	None
RMC04^*^	500	109.01	12.5	88	None
RMC96^★^	3000	109.4	8.5	52	None

^§^Estimated values from supplementary information in ref. ^14^.

^†^From ref. ^23^.

^‡^From ref. ^13^.

*From ref. ^10^.

^★^Results for a-Ge from ref. ^9^.

### Comparison with total-energy-based models

In order to demonstrate the efficacy of the data-driven CMC19 approach over conventional approaches, using total-energy and forces, we now compare the results with an array of models from molecular dynamics and Monte Carlo simulations. While there exist a plethora of such models in the literature, we confine ourselves to few high-quality *a*-Si models that can produce accurate structural, electronic, and vibrational properties of *a*-Si. To this end, we chose four representative models for comparison: (1) a classical molecular-dynamics (MD) model^25^ based on the modified Stillinger-Weber potential^26^; (2) an MD model based on machine-learning (ML) potential^27^; (c) an *ab initio* MD model^28^; and (d) a model obtained from using the Wooten-Winer-Weaire (W3)^29^ algorithm, modified by Barkema and Mousseau (BMW3)^30^. The last approach can produce large 100% defect-free configurations of *a*-Si, and it is often considered as the benchmark for high-quality *a*-Si models from simulations. These four models will be collectively referred to as *total-energy-based* (TEB) models.

Table 2 presents an overview of structural properties of the TEB models, along with the CMC19 models of size from 216 to 1000 atoms before and after *ab initio* relaxations. Here, the results for CMC19, BMW3 and SWMD models were all averaged over five (5) independent configurations. It is apparent that the structural quality of the CMC19 models is on a par with the BMW3 model, and that the former are considerably better than those derived from AIMD and MLMD models, as far as the 4-fold atomic coordination of the models are concerned. This observation equally applies to the electronic quality of the models, which is reflected in the size of the electronic gap. Considering the long simulation time (≥20 ns) needed to produce the SWMD models^25^ and the complexity associated with generating a machine-learning potential^27^ for *a*-Si, it is evident that the CMC19 approach produces *a*-Si models par excellence, using diffraction data and constraints only. Not only do the CMC19 models show the presence of an essentially clean gap in the electronic spectrum, but also the size of the gap, *E*_*g*_, matches closely with the value obtained from the BMW3 model. A relatively small value of *E*_*g*_ for the 1000-atom CMC19 model can be attributed to the presence of 0.9% coordination defects and a somewhat larger value of Δ*θ*, compared to its BMW3 counterpart, which affect the band-edge states.

**Table 2:** Comparison of CMC19 models with the best available models (of *a*-Si) in the literature. Symbols have the same meaning as in Table 1. *E*_*g*_ and bond angles are expressed in the unit of electron-volt (eV) and degree, respectively.

Model	N	〈*θ*〉	Δ*θ*	*c*_4_	*c*_2_	*c*_3_	*c*_5_	*E*_*g*_
***Unrelaxed*** **CMC19 models**								
CMC19	216	109.12	10.68	99.07	0	0.93	0	1.14
CMC19	300	109.14	10.56	99.33	0	0.67	0	1.06
CMC19	512	109.19	10.70	98.44	0	1.56	0	0.88
CMC19	1000	109.13	11.15	99.10	0.30	0.60	0	0.56
***Ab-initio-relaxed*** **CMC19 models**								
CMC19	216	109.08	10.95	100	0	0	0	1.18
CMC19	300	109.16	10.19	99.33	0	0.67	0	1.39
CMC19	512	109.14	10.61	99.22	0	0.78	0	1.09
CMC19	1000	109.08	11.43	99.10	0.30	0.40	0.20	0.76
***Total-energy-based*** **models**								
BMW3^§^	512	109.14	10.36	100	0	0	0	1.32
SWMD	512	109.27	9.12	99.22	0	0.39	0.39	1.01
MLMD^†^	512	109.19	9.69	98.44	0	0.78	0.78	NA
AIMD^‡^	64	108.32	15.5	96.60	0	0.20	3.20	NA

^§^From ref. ^30^.

^†^From ref. ^27^.

^‡^From ref. ^28^.

**Figure 1:**
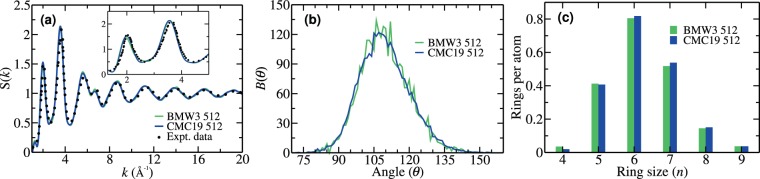
Comparison of structural properties of *a*-Si from CMC19 and BMW3 models. (**a**) The structure factor of a 512-atom CMC19 model (blue) and a 512-atom BMW3 model (green). Experimental data (black) correspond to as-deposited samples from ref. ^31^. An enlarged view of the first two peaks is shown in the inset. (**b**) The bond-angle distribution, *B*(*θ*), for a CMC19 model (blue) and a BMW3 model (green) of identical size. (**c**) The statistics of irreducible rings for CMC19 and BMW3 models. See text for details. The results presented here in (**a**–**c**) are all averaged over five (5) independent configurations.

Figure 1(a) presents the structure factor of *a*-Si obtained from a 512-atom CMC19 model. For comparison, the corresponding structure factor from a BMW3 model of identical size and from experiments^31^ are also included in Fig. 1(a). Owing to the form of the objective function in Eq. (1), it is not surprising that the structure factor from the CMC19 model matches closely with the same quantity from the BMW3 model and the experimental data from as-deposited samples in ref. ^31^. Likewise, the bond-angle distribution in Fig. 1(b), with a root-mean-square (RMS) bond-angle width of 10.61°, is also found to match very well with its BMW3 counterpart, which is characterized by an RMS width, Δ*θ*, of 10.36°. Assuming that the bond-angle distribution can be approximated by a Gaussian function, this value corresponds to a full width at half maximum (FWHM) of 23–25°, which closely matches with the measured value of the FWHM of about 22–23°, from Raman spectroscopy^32^. It is notable that none of the CMC19 models has any 60° bond angles, and the vast majority of the bond angles are narrowly distributed, between 90° and 135°, as shown in Fig. 1(b). Together with the structure factor, the bond-angle distribution and its RMS width play an important role in determining the structural quality of tetrahedral amorphous semiconducting networks.

**Figure 2:**
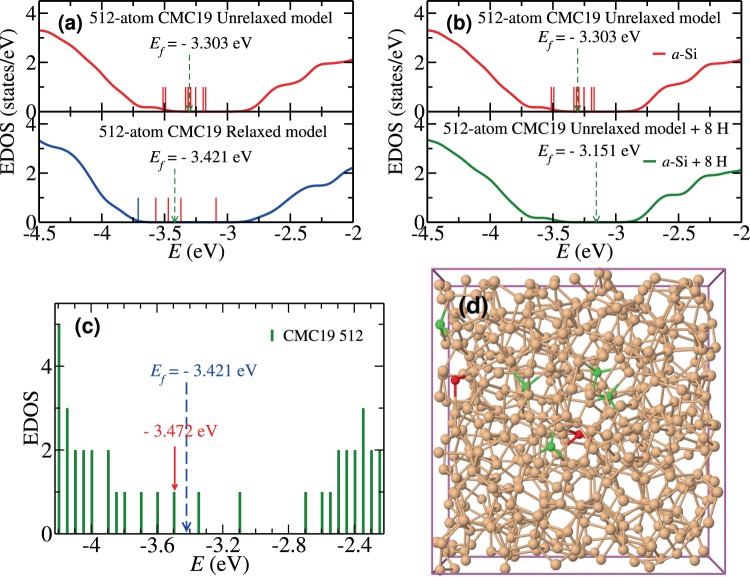
Electronic density of states (EDOS) of CMC19 *a*-Si models near the band gap. (**a**) The EDOS of *a*-Si before (upper panel) and after (lower panel) *ab initio* relaxation of a 512-atom CMC19 model. A few defect states (red) and a band-edge state (blue) are shown as vertical lines in the gap region. (**b**) The formation of a clean electronic gap in the unrelaxed 512-atom CMC19 model via hydrogenation. The upper and lower panels correspond to the EDOS before and after H passivation, respectively. (**c**) A defect state at −3.472 eV and (**d**) the associated dangling bonds (i.e., 3-fold-coordinated sites shown in red color) in real space. The other contributing sites, with 4-fold atomic coordination, are indicated in green color.

Having discussed the local structural properties of the models, we shall now address to what extent the atomistic structures on the medium-range length scale resemble or differ from those of the BMW3 models. This is particularly relevant for CMC19 models, as the constraint functions in Eq. (1) do not carry any information beyond the first nearest-neighbor distance, and the experimental two-body correlation data, *F*_*ex*_(r; **R**), may not be sufficient to include the characteristic structural properties associated with higher-order atomic correlation functions. We address this by examining the topological connectivity of the networks, which involves irreducible rings of various sizes and the dihedral-angle distribution. Intuitively speaking, given the local tetrahedral character of the network, the distribution of *n*-member rings (n ≥ 4) provides some information about the atomistic structure on the medium-range length scale, whereas the dihedral-angle distribution should exhibit some characteristic features of a (reduced) 4-body correlation function. Figure 1(c) shows the distribution of irreducible rings, of sizes from *n* = 4 to *n* = 9, from a CMC19 model and a BMW3 model of size 512 atoms. It is apparent that, despite the absence of a total-energy functional, the CMC19 models exhibit similar topological connectivity as that for the BMW3 models. See supplementary information for a further discussion on the structural characterization of the models.

### Electronic properties of amorphous silicon from CMC19

While structural properties of model networks provide a wealth of atomistic information, the most compelling evidence of the accuracy and the reliability of a purely information-driven method, and the resulting structures therefrom, come from its ability to produce the correct electronic properties, obtained without the use of any total-energy functionals during structural formation. For predictive simulations of complex disordered materials using experimental data and information only, it is of crucial importance to establish the ‘thermodynamic’ stability of the models. An effective inverse approach must thus be able to produce structural configurations that sit close to a stable local minimum of an appropriate total-energy functional. However, these primal issues were ignored in earlier RMC studies by overemphasizing the importance of pair-correlation or structure-factor data, and the resulting effects on the three-dimensional structure associated with these one-dimensional data. Although a few RMC studies^10,13,14,21^ on *a*-Si/*a*-Ge did report a narrow bond-angle distribution or a high concentration of 4-fold atomic coordination, those results were obtained at the expense of one or the other. Consequently and unsurprisingly, none of the models from previous studies could produce a gap in the electronic spectrum, either due to a high concentration of coordination defects or due to the presence of considerable disorder in the bond-angle distribution of atoms, leading to either a gapless electronic spectrum or a spectrum with a pseudo-gap. It is therefore necessary to examine the electronic density of states (EDOS) of the unrelaxed CMC19 models in order to establish that the models are indeed stable prior to structural relaxation and that they exhibit an electronic gap in the spectrum (see Table 2).

Figure 2(a) shows the EDOS of a 512-atom CMC19 model near the band-gap region before (upper panel) and after (lower panel) *ab initio* structural relaxation. Here, we employed the first-principles density-functional code Siesta^33^, using double-zeta basis functions and the generalized-gradient approximation (GGA)^34^. It is evident that the unrelaxed CMC19 model can produce the correct electronic density of states in the vicinity of the electronic-gap region. Aside from a few mid-gap defect states, indicated as vertical red lines in Fig. 2(a,b), the CMC19 model produces a clean spectral gap that constitutes a major outcome of this study, previously unreported in the literature. A typical defect state (at −3.472 eV) near the Fermi level is shown in Fig. 2(c). The state is primarily originated from two dangling bonds (DBs) in real space, which are shown in Fig. 2(d) in red color. A few neighboring atoms with secondary contribution are also indicated in green color. A few defect states that appeared in the band-gap region can be readily passivated by adding a minute amount of hydrogen. This is illustrated in Fig. 2(b). The lower panel of Fig. 2(b) depicts the hydrogen-passivated EDOS for the unrelaxed, i.e., *static* 512-atom CMC19 model, obtained by placing eight (8) H atoms near Si dangling bonds. Hydrogen atoms were so added that the local tetrahedral arrangement of the DBs was minimally perturbed and the silicon-hydrogen bond length was restricted to a distance of 1.55 ± 0.05 Å. The passivation of the Si DBs by H atoms in the static 512-atom CMC19 model yields a clean electronic gap of size 0.85 eV, without *ab initio* total-energy relaxations, which leads to a high-quality model of *a*-Si:H with 1.54 at. % of hydrogen. This procedure can be readily generalized to obtain device-quality models of *a*-Si:H, with a varying concentration of hydrogen. Thus, the CMC19 approach presented in this paper is equally effective in producing models of hydrogenated amorphous silicon.

**Figure 3:**
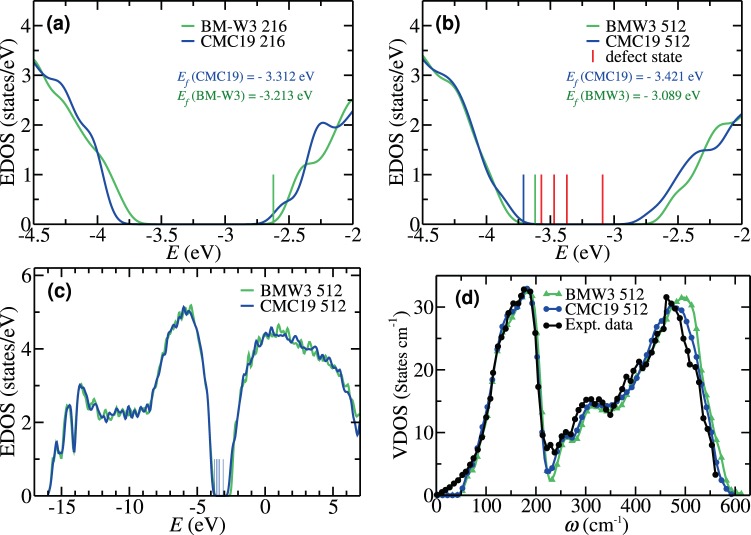
Electronic and vibrational densities of states for *a*-Si from CMC19/BMW3 models. (**a**) A 216-atom CMC19 model and its BMW3 counterpart. (**b**) A 512-atom CMC19 model and a 512-atom BMW3 model, along with a few defect states (red) and band-edge states (blue and green). (**c**) The full EDOS of *a*-Si for a 512-atom CMC19 model and a BMW3 model. The vertical lines (blue) in the gap region indicate defect states. (**d**) The VDOS from a 512-atom CMC19 and a 512-atom BMW3 model. Experimental data (black) shown here are from inelastic neutron-scattering measurements^35^.

Since the EDOS near the valence and conduction bands is highly susceptible to disorder and the presence of coordination defects, particularly dangling bonds and the RMS width of bond angles, the presence of a clean gap in the electronic spectrum is often considered as the most stringent test of the electronic quality of a model. Until recently, with the exception of the BMW3 models, there exist no models that can exhibit a clean spectral gap in the electronic density of states. It is remarkable that the information-driven CMC19 models with 216 atoms and 300 atoms exhibit a pristine gap, and those with 512 atoms and 1000 atoms yield a nearly clean gap, in the EDOS [see Fig. 3(a–c), Table 2 and additional results as supplementary information], even though no total-energy functional was employed during the course of minimization of the objective function. A comparison of *E*_*g*_ values in Table 2, obtained before and after total-energy relaxation of the CMC19 models, firmly establishes that the data-driven CMC19 models are energetically stable and the latter indeed represent the correct structural solution that one strives to obtain from *ab initio* MD simulations of *a*-Si.

The full EDOS for the 512-atom CMC19 model is presented in Fig. 3(c), along with the results from the corresponding BMW3 model. The EDOS from these two models practically matches point-by-point, except for a few points near the conduction-band edge, as shown in Fig. 3(b). This minor deviation can be partly attributed to a slightly higher value of Δθ for the 512-atom CMC19 model compared to its BMW3 counterpart. The value of the electronic gap, *E*_*g*_, reported in Table 2, was computed by ignoring the defect states in the electronic spectrum. This was achieved by examining each of the states in the vicinity of the gap region and determining whether it originated from coordination defects in real space or not (see Fig. 2(d) and supplementary information). Similarly, the vibrational excitations from a 512-atom CMC19 model, which crucially depend on the local atomic structure, have been found to agree well with those from a BMW3 model of equivalent size and experiments. This is evident from Fig. 3(d), where the vibrational density of states (VDOS) for a 512-atom CMC19 model, a 512-atom BMW3 model, and experimental data from inelastic neutron-scattering measurements from ref. ^35^ are presented for comparison. The computed values of the VDOS, obtained from the harmonic approximation of dynamical matrices, match very well with those from experiments and the BMW3 model. A small deviation below 50 cm^−1^ can be attributed to finite-size effects. Assuming a linear dispersion relation, which (generally) holds in the limit *k* → 0, one may expect that the normal-mode frequencies below a characteristic frequency (*ω*_*c*_ ∝ *k*_*c*_) would be absent in finite-size models, owing to the presence of a lower wavevector cutoff (*k*_*c*_ ≈ $\frac{4\pi }{L}$. Additional minor deviations from experimental data near 225 cm^−1^ may have originated from the application of the harmonic approximation in the computation of the VDOS.

**Figure 4:**
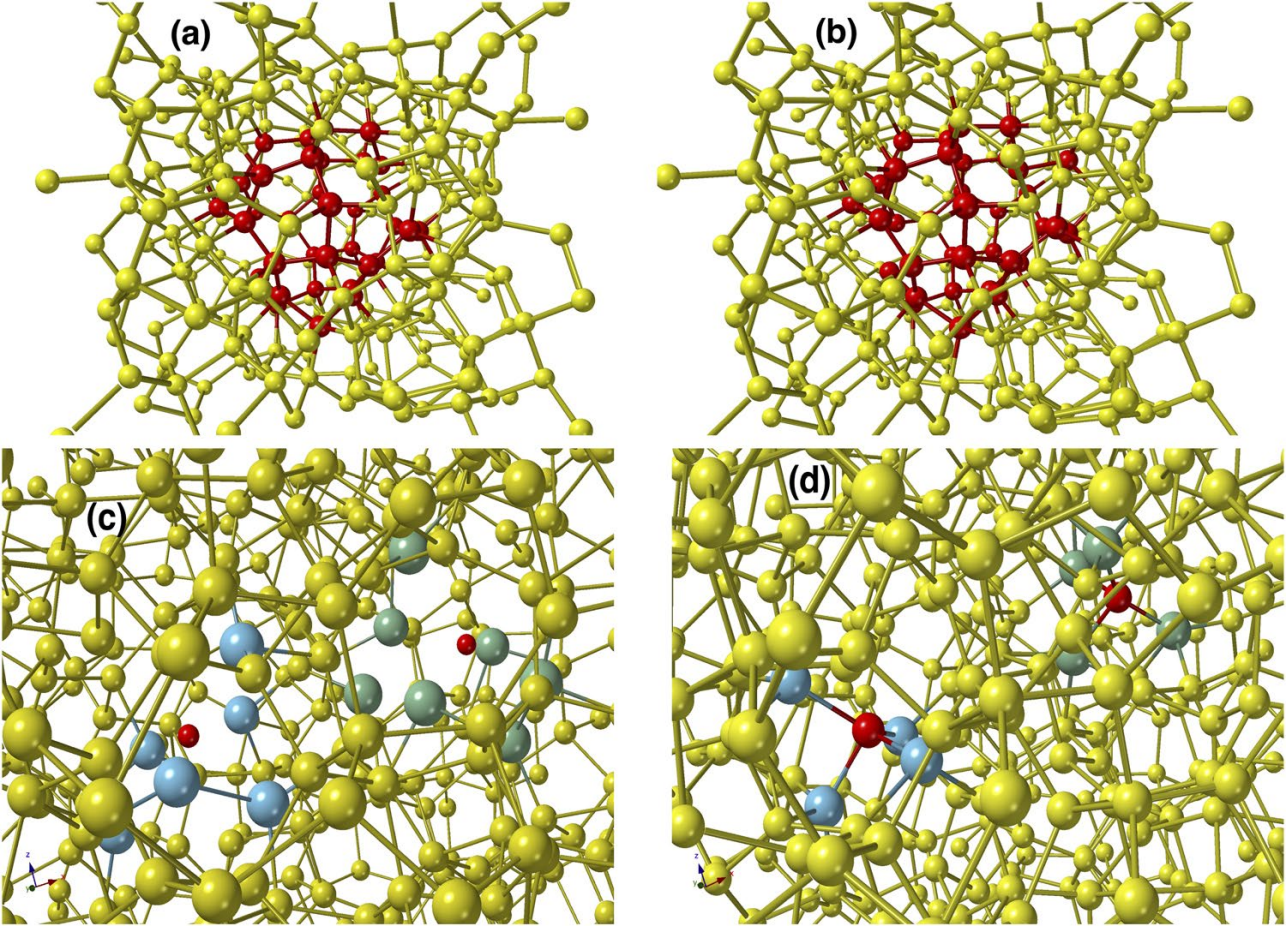
An illustration of the formation of voids and vacancies in *a*-Si via the CMC19 approach. (**a**) A 300-atom CMC19 model with a void of radius 4 Å at the center of the model. The atoms on the void surface (of width 2 Å) are shown in red color. (**b**) The same model after *ab initio* total-energy relaxation, showing the structural stability of the void. (**c**) A 512-atom CMC19 model with two monovacancies, separated by a distance of 9 Å. The blue (left) and green (right) colors indicate the atoms within the region of 4 Å from the center of the respective vacancy, which is indicated by a small (hypothetical) red sphere. (**d**) The *ab-initio*-relaxed 514-atom model obtained by adding a silicon atom (red) at the center of each vacancy in (**d**). The formation of a local 4-fold-coordinated network at/near the vacancy sites establishes that the vacancy region corresponds to a monovacancy site. The missing Si atoms are shown in red color.

### Microstructure of realistic samples of *a*-Si: Modeling vacancies and voids via CMC19

In the preceding sections, we have discussed the structural, electronic and vibrational properties of *a*-Si, obtained from almost idealized CRN models. As discussed in the Method section, the microstructure of realistic samples of *a*-Si prepared in laboratories can be rather complex, depending upon the growth conditions, the method of preparation, and the history of the samples, and may not be described adequately using the simplistic CRN model of *a*-Si. However, unlike conventional MD and MC methods, the CMC19 approach presented here can effectively address a number of microstructural properties of *a*-Si, such as voids and vacancy defects, by incorporating appropriate structural information. To this end, we now discuss these properties by analyzing two *a*-Si models, obtained from using the CMC19 approach, which are characterized by the presence of voids and vacancy-type defects observed in laboratory-grown samples of *a*-Si.

The results for a 300-atom model and a 512-atom model, obtained from using the modified objective function in Eq. (2) [see Method section], confirm that the approach is very useful to describe microstructural properties of *a*-Si, starting from a random configuration. Figure 4(a,b) show the network structure of a 300-atom CMC19 model with a single void of radius of 4 Å before and after *ab initio* total-energy relaxation, respectively. The silicon atoms on the surface of the void, between radii 4 Å and 6 Å, are shown in red color for visual clarity. The structural stability of the void (i.e., void-surface atoms) is reflected in Fig. 4(b), which is found to remain intact during total-energy relaxations. This observation demonstrates that one may include inhomogeneities or voids of varying sizes in the amorphous matrix of *a*-Si/*a*-Ge so that the void-volume density is consistent with the experimentally observed value of 0.02–0.3%, depending upon the method of preparation and conditions. The electronic density of states of the 300-atom CMC19 model with a void at its center is provided as electronic supplementary information.

The vacancy-type defects can also be incorporated in a similar manner by producing an array of microvoids of radius 3–4 Å. This is illustrated in Fig. 4(c,d) by adding two isolated monovacancies in a 512-atom CMC19 model during the course of the CMC19 simulations. The vacancy regions are shown in Fig. 4(c) by light blue (left) and green (right) atoms, which are at distance of up to 4 Å from the center of the respective vacancy. The vacancy centers are indicated in Fig. 4(c) by two small (hypothetical) red spheres, which are separated by a distance of 9 Å. The fact that these regions truly represent stable monovacancies, and not microvoids, can be readily verified by placing a silicon atom at the center of each vacancy and relaxing the resulting (512 + 2)-atom model using *ab initio* total-energy functionals. It is evident from Fig. 4(d) that the addition of two Si atoms passivated the pair of vacancies by restructuring the local regions at or near the vacancy sites, which led to the formation of a defect-free 4-fold-coordinated local network. The atoms within a radial distance of 3.2 Å from the vacancy centers are shown in light blue and green colors. Thus, the pair of isolated vacancies in Fig. 4(c) can be viewed as originating from the missing two Si atoms, shown in Fig. 4(d) in red color, which are separated by a distance of 9.17 Å in the *ab initio*-relaxed model. The presence of such 1–2% vacancy-type defects in annealed samples of *a*-Si was suggested by Laaziri *et al*. in ref. ^31^ in order to explain the observed value of the average coordination number of 3.88 from X-ray diffraction. It may be noted that one often justifies the presence of too many coordination defects in MD or other models by quoting the average coordination number of 3.88, obtained from the RDF of annealed *a*-Si samples. However, a direct comparison with model calculations would be incorrect as it leads to an isolated dangling-bond density of about 12%, which is neither compatible with ESR and IR measurements nor with the observed electronic density of states of *a*-Si. A more plausible explanation is the presence of vacancy-type defects and microvoids in bulk *a*-Si that leads to an average coordination number of 3.88 in annealed samples. In summary, using an augmented form of the objective function, the data-driven CMC19 methodology not only provides a means to generate accurate structural models of *a*-Si/*a*-Ge but also to include microstructural properties of the materials observed in experiments without employing any total-energy functionals and forces.

## Conclusions

In this paper, we present a purely data-driven multiobjective constraint Monte Carlo (CMC19) approach that can produce accurate structural models of tetrahedral amorphous semiconductors without employing a total-energy functional but using diffraction data and local structural information only. By posing the material-structure determination as an inferential problem and addressing the problem as an optimization program, we have shown that the problem can be solved by inverting diffraction data to generate a three-dimensional structural solution, using Monte Carlo methods. Owing to its dependence on local information, the approach can be implemented efficiently using an order-*N* algorithm for the evaluation of the objective function of a system consisting of *N* atoms. An examination of the unrelaxed CMC19 models and their *ab-initio*-relaxed counterparts shows that the former sits very close to a stable local minimum of a quantum-mechanical total-energy functional, indicating the thermodynamic stability of the information-driven CMC19 models. The hallmark of this new constraint-driven order-*N* approach is that it has the ability to produce 100% defect-free model configurations of amorphous silicon, as exemplified by a 216-atom model. Comparisons of structural, topological, electronic and vibrational properties of the models with those from experiments revealed that the CMC19 models are structurally, topologically and electronically accurate. The salient features of the models include a narrow bond-angle distribution, with an RMS deviation of 9–11.5°, and an ultra-low defect concentration below 1%, which enables the models to exhibit a clean electronic gap of size 0.8–1.4 eV. To our knowledge, none of the RMC or RMC-derived inverse and hybrid models in the literature so far, which employ diffraction data and a total-energy functional, can produce the aforementioned structural and electronic properties as accurately as the CMC19 models. The information-based CMC19 approach presented here not only can produce overall structural and electronic properties but also the microstructural properties of realistic samples of *a*-Si from experiments, such as voids and vacancy-type defects, which cannot be addressed directly using currently available computational methods. This observation heralds the resolution of the long-standing problem of uniqueness in the structural determination of tetrahedral amorphous semiconductors via inversion of diffraction data, in particular *a*-Si, without employing a total-energy functional. The study demonstrates that information-driven inverse approaches not only can enhance existing methodologies for modeling disordered materials, but also offer a directional step change in materials computation and radically different approaches to the structural determination of disordered materials, based on an information paradigm.

## Methods

In our multiobjective constraint optimization approach, we begin with an objective function, *χ*^2^(**R**), which includes information from experimental diffraction data and a set of structural constraints,

1 $${\chi }^{2}({\bf{R}})={\sum _{i=1}\left[\frac{{F}_{ex}({q}_{i})-{F}_{c}({q}_{i};{\bf{R}})}{\sigma ({q}_{i})}\right]}^{2}+\mathop{\sum }\limits_{l=1}^{{l}_{m}}{\lambda }_{l}{C}_{l}({\bf{R}}),$$

where ${F}_{c}({q}_{i};{\bf{R}})$ correspond to simulated diffraction data, either in wavevector space (*q* = *k*) or in real space (*q* = *r*), obtained from a distribution of atoms **R**, *σ*(*q*_*i*_) is the error associated with experimental data, *F*_*ex*_(*q*_*i*_), and *C*_*l*_*s* are a set of *l*_*m*_ constraints, providing additional information on the structural properties of the solid. The coefficients *λ*_*l*_ are weights, which determine the relative strength of each constraint in Eq. (1). To incorporate constraint information, we prescribe a convex function, *C*_*l*_ = (*f*_*l*_(**R**)  −  ${f}_{l}^{0}$)^2^, where *f*_*l*_ represents a structural variable, associated with a configuration **R**, and ${f}_{l}^{0}$ corresponds to the same for a true but unknown solution **R**_0_. Our goal is to determine an accurate structural solution **R**′_0_, which is *sufficiently* close to **R**_0_, by simultaneously minimizing *C*_*l*_(**R**) and fitting the computed structure-factor or pair-correlation data with their experimental counterpart. Two structural configurations, **R**_0_ and **R**′_0_, are considered to be sufficiently close when a minimal number of physical observables, derived from these configurations, are found to be almost identical to each other and that these observables can be employed to define a structure uniquely. For amorphous tetrahedral semiconductors, the local chemistry suggests, *f*_1_ = 〈*θ*〉, *f*_2_ = Δ*θ* and *f*_3_ = *c*_4_, where 〈*θ*〉, Δ*θ* and *c*_4_ represent the average bond angle, the root-mean-square (RMS) deviation of *θ* and the percentage of 4-fold-coordinated atoms in **R**, respectively. The parameters *λ* play an important role in the optimization of the objective function, *χ*^2^(**R**), which largely determine the evolution of an approximate solution, **R**′_0_, via important sampling of the objective function in high-dimensional space, using Monte Carlo optimization. One frequently chooses *λ*_*l*_, by trial and error, so that both the objective function and its constraint components can be simultaneously optimized during the CMC runs. It may be noted that, in the presence of conflictive constraints, several pareto-optimal sets of *λ*_*l*_ exist, which suffice to generate a set of good structural solutions. The intention here is not to find an optimal set of λ values, which is somewhat analogous to developing a three-body potential, but to obtain accurate structural solutions consistent with given data sets. To obtain a close structural solution, **R**′_0_, the inversion procedure was implemented by minimizing Eq. (1), using simulated annealing techniques. An initial random configuration, consisting of *N* Si atoms in a cubical box of length *L*, was generated so that the density of the model corresponds to the experimental density, 2.25 g/cm^3^, of *a*-Si, and no two atoms were at a distance less than 2 Å. The latter was enforced throughout the course of simulations to maintain an excluded volume of radius 1 Å surrounding each Si atom. By choosing *F*_*c*_(*q*;**R**) as the pair-correlation function, the Metropolis algorithm was employed to accept or reject a trial move, **R**_*i*_ → **R**_*f*_, following the Metropolis acceptance probability P = min [1, exp(−*β*Δ*χ*^2^)], where Δ*χ*^2^ = *χ*^2^(**R**_*f*_) − *χ*^2^(**R**_*i*_) and *β* (= 1/*k*_*B*_T) is an optimization parameter or the inverse temperature of the system. The system was initially equilibrated at a hypothetical temperature of 310 K for 10^5^ Monte Carlo steps (MCS), which was followed by linear cooling of the system from 310 K to 10 K, in steps of 25 K, for every 10^5^ MCS at each temperature. Since the objective function in Eq. (1) is dimensionless, the temperature of the system is fictitious. Thus, it is not necessary to define temperature via *β* = $\frac{1}{{k}_{B}T}$, and one may directly proceed to choose a suitable value of *β* to optimize the objective function. Our choice of *β* assumes a hypothetical energy scale to provide an intuitive understanding of the role of *β* in Monte Carlo parlance of the optimization of a total-energy functional via simulated annealing. Throughout this work, we have used a value of 8.625 × 10^−5^ K^−1^ for *k*_*B*_.

The MC procedure was repeated, from 110 K to 1 K, for at least 5 or more cycles, until the RMS width of the bond-angle distribution and the concentration of coordination defects reduced to about 11° and 1%, respectively. The simulations were performed by moving one atom at a time and the maximum atomic displacement was restricted to 0.15–0.2 Å in order to keep the acceptance rate as high as possible. The advantage of moving of one atom (or a few atoms) at a time is that it provides a means to move atoms in the specific regions of interest and to develop an order-*N* optimization algorithm for the computation of Δ*χ*^2^, associated with the displacement of one atom (or a few atoms)^36^. The linear scaling was achieved by updating an initially generated pair-correlation function and the list of nearest neighbors of a (few) selected atom(s) as the simulation proceeds. This was particularly necessary to address systems larger than 500 atoms. A detailed description of the order-*N* method will be presented elsewhere. Depending upon the temperature, the size of the system, and the magnitude of the maximum possible displacement of an atom during MC simulations, the acceptance rate (of the MC moves) was found to vary from 25% to 50%. After several trial runs, we settled for two sets of *λ* values. For 1000-atom models, we used *λ*_1_ = 2/3, *λ*_2_ = 4/3, and *λ*_3_ = 2/3, whereas the corresponding values for 216-atom, 300-atom, and 512-atom models are given by 1/3, 2/3, and 1/15, respectively. Given the pareto-optimal nature of the problem, it is possible to employ a different set of *λ* values in order to incorporate constraints with a varying strength. To conduct the configurational averaging of physical properties and to demonstrate the reproducibility of results from our method, ten independent configurations for each system size were generated and studied in our work. Since the results from simulated annealing techniques may often vary, depending upon the cooling protocol used in simulations, we also employed a second cooling scheme with an exponential decay of temperature to examine its possible effect on the quality of the final solutions. The results from the exponential cooling scheme were found to be similar to those from the linear cooling scheme.

Having provided an ansatz for reconstructing the three-dimensional structure of tetrahedral amorphous semiconductors, without using a total-energy functional, via the inversion of experimental data in the presence of structural constraints, we now address the realistic modeling of microstructure of *a*-Si/*a*-Ge observed in experiments. The use of a minimal number of constraints leads to a natural solution as continuous random networks (CRN). However, it is widely acknowledged that a CRN model cannot provide a comprehensive description of *a*-Si/*a*-Ge, and the microstructure of *a*-Si/*a*-Ge from experiments may considerably vary from sample to sample, depending upon the method of preparation, history of the samples, preparation conditions, etc. Thus, while a CRN model provides for the most part a correct description of some key structural and electronic properties of *a*-Si, it cannot produce many important characteristics of laboratory-grown samples, such as the distributions of defects and microvoids, local inhomogeneities, and the presence of different topologically-connected regions^37^, observed in experiments. These microstructural properties are often addressed by introducing ad hoc structural measures or changes in CRN models. For example, the results from fluctuation electron microscopy (FEM)^37^ are often explained either by including small grains of paracrystals^37^ or nanometer-size voids^38^, density fluctuations by implanting vacancy-type defects^39–41^, and extended inhomogeneities by voids^42^ in CRN models. Although these ad hoc measures are found to be largely successful in describing the microstructural properties of *a*-Si and *a*-Ge, associated with various methods of preparation and experimental conditions, they cannot nevertheless replace the need for a systematic data-driven method. While machine-learning (ML) approaches appear to be a promising route to address some of these issues, their success crucially depends upon the availability of suitable training data and the ability of the underlying ML model to obtain an optimal set of learning parameters, which involves solving an optimization program under a different guise. Likewise, the majority of conventional MD simulations tend to produce too many dangling-bond defects (≥5%), which often render the MD models unsuitable without further treatment, as far as the defect density and electronic density of states (EDOS) are concerned. By contrast, the information-based approaches can be generalized by directly including appropriate microstructural constraints, guided by experimental information or data, to develop models with realistic microstructural properties as observed in experiments. For example, to generate *a*-Si models with a given void-volume density, or vacancy-type defects, one may include an additional term *g*(**r**;***R***_*v*_) to *χ*^2^(**R**) and write,

2 $${\chi {\prime} }^{2}({\bf{R}})={\chi }^{2}({\bf{R}})+{\sum _{k}{{\gamma }}_{{k}}|g({\bf{r}}-{{\bf{r}}}_{{\bf{k}}};{{R}}_{{v}})|}^{2},$$

where $g({\bf{r}}-{{\bf{r}}}_{{\bf{k}}};{{R}}_{{v}})$ is a function that produces a void, or a vacancy, at **r** = **r**_**k**_ of linear size *R*_*v*_. Equation (2) can be viewed as a regularization of the original objective function in (1), which can be optimized for a suitable value of γ_*k*_ and g(**r**, *R*_*v*_). The shape and size of the voids can be approximately controlled by choosing a suitable functional form of $g({\bf{r}}-{{\bf{r}}}_{{\bf{k}}};{{R}}_{{v}})$. For example, a spherical void or vacancy region can be constructed by using an inverse quadratic function or a Fermi function, characterized by an appropriate shape parameter, which determines the boundary region of a void/vacancy from the rest of the network. The efficacy of this approach to describe the microstructure of *a*-Si is illustrated here by providing two examples involving voids and vacancy-type defects.

## Supplementary information

Supplementary information.

## Data Availability

The authors declare that the data supporting the findings of this study are available within the paper and the atomistic models used to generate the data are provided as electronic supplementary information.
