# Supplementary material for: Disorder by design: A data-driven approach to amorphous semiconductors without total-energy functionals
Source: Sci Rep. 2020 May 8;10:7742. doi: 10.1038/s41598-020-64327-3 (PMC7210951; doi:10.1038/s41598-020-64327-3)
Supplement: Supplementary file 1 — Supplementary information. [file 41598_2020_64327_MOESM1_ESM.pdf]

Supplementary Information for

Disorder by design: A data-driven approach to amorphous semiconductors without total-energy functionals

Dil K. Limbu,[1] Stephen R. Elliott,[2] Raymond Atta-Fynn,[3] and Parthapratim Biswas[1]

[1]*Department of Physics and Astronomy,*

The University of Southern Mississippi, Hattiesburg, Mississippi 39406, USA

[2]*Department of Chemistry, University of Cambridge,*

Cambridge CB2 1EW, United Kingdom

[3]*Department of Physics, The University of Texas at Arlington, Texas 76019, USA*

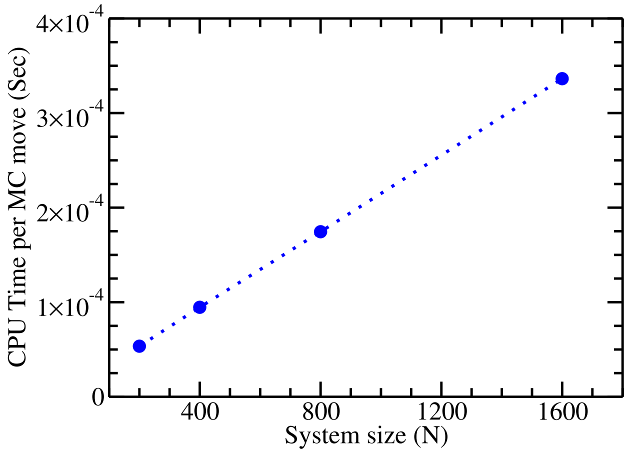

Figure S1. The variation of CPU time (in seconds) with system size N for a single MC move, associated with the evaluation of the objective function (see Eq. 1). The CPU time is found to scale linearly with N.

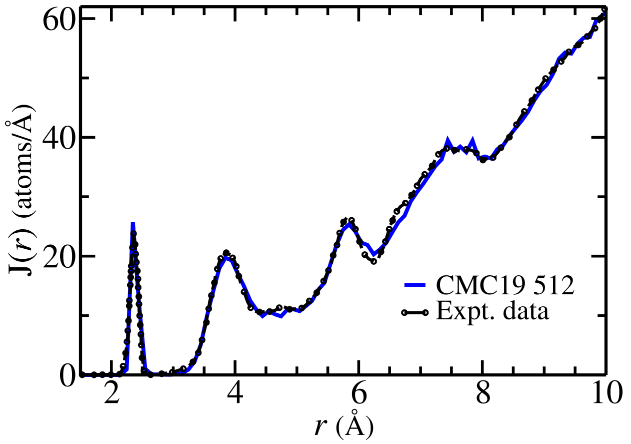

Figure S2. The radial distribution function (RDF), $J(r)$, for the 512-atom CMC19 model and the experi-mental data obtained from as-deposited samples of *a*-Si reported in Ref. 31.

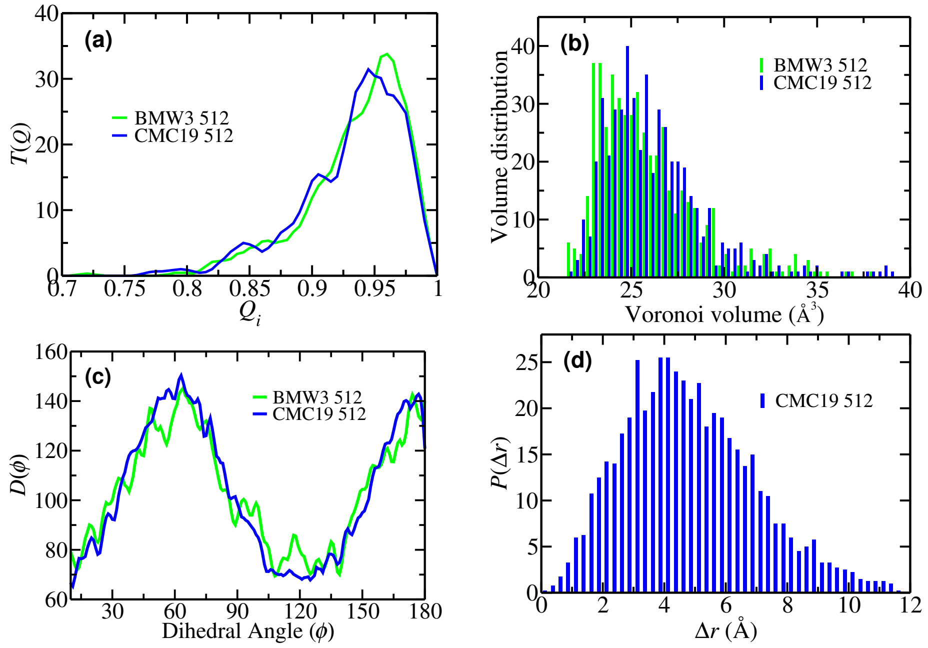

Figure S3. Comparison of additional structural properties of CMC19 models with BMW3 models. (a) The distribution, $T(Q_i)$, of the local tetrahedral order parameter, Q_i, for a CMC19 model, along with its BMW3 counterpart of identical size. (b) The distribution of Voronoi volumes for a 512-atom CMC19 model and its BMW3 counterpart. (c) The dihedral-angle distribution, $D(\phi)$, from a CMC19 model and its BMW3 counterpart, showing the characteristic dihedral peaks for tetrahedral ordering near 60° and 180°. (d) The distribution of the resultant displacements of atoms during CMC19 simulations for a 512-atom model.

ADDITIONAL DISCUSSION OF FIGURE S3

In an effort to characterize the models further, and to compare the CMC19 models with their corresponding BMW3 counterparts, we have examined the local degree of tetrahedrality, the voronoi-volume distribution of the atoms, and the dihedral-angle distribution of the amorphous networks in Fig. S3(a)-(c). While the average bond angle, $\langle\theta\rangle$, and its width, $\Delta\theta$, provide an overall measure of the tetrahedral character of a network, the tetrahedral order parameter (TOP), Q_i, at a site i, and its distribution, $T(Q_i)$, gives a precise local measure of the degree of tetrahedrality and its deviation from the ideal TOP value of unity. The local TOP can be written as,[24]

$$Q_i = 1 - \frac{3}{8}\sum_{\{jik\}}\left(\cos\theta_{jik} + \frac{1}{3}\right)^2, \;\; \langle Q\rangle = \frac{1}{N}\sum_i Q_i,$$

where the symbol $\{jik\}$ stands for the sum over all possible nearest-neighbor angles subtended at the site i. It has been observed that the electronic quality of a model depends on $T(Q_i)$, $\Delta\theta$, and the concentration of coordination defects.[26] In particular, the local TOP is sensitive to the presence of floating bonds (i.e., 5-fold-coordinated atoms), and the existence of dangling bonds (i.e., 3-fold-coordinated atoms) is readily reflected in the electronic density of states near the Fermi level as defect states. A comparison of $T(Q_i)$ in Fig. S3(a) clearly establishes the similarities between an CMC19 model and a BMW3 model, as far as the degree of local tetrahedrality is concerned. This observation is consistent with the statistics of n-fold coordination numbers, c_n, given in Table 2. A similar observation follows from a comparison of the voronoi-volume distributions of CMC19 and BMW3 models as seen in Fig. S3(b). The marked similarities between the CMC19 and BMW3 models, obtained from two distinctly different approaches, are indeed remarkable in view of the fact that the former do not include any information from a total-energy functional.

Likewise, the dihedral angles, involving a chain of four consecutive nearest neighbors, are also found to be distributed in a similar way for the CMC19 and BMW3 models in Fig. S3(c). These results collectively suggest that the two models are atomistically similar as far as the topological connectivity and the dihedral angles, involving the first few neighbors on the length scale of 3–6 Å, are concerned. In this context, it is appropriate to note that, during CMC19 simulations, structural formation takes place via the rearrangement of the great majority of atoms (about 92%) within the first four neighboring shells, over a radial distance of up to 8 Å. This is evident from Fig. S3(d), where the distribution, $P(\Delta r)$, of the resultant atomic displacements, Δr, is plotted. Here, $\Delta r = |\mathbf{r}_f - \mathbf{r}_i|$ and $\mathbf{r}_i$ and $\mathbf{r}_f$ indicate the initial and final positions of an atom at the beginning and end of simulations, respectively. In other words, Δr indicates the magnitude of the resultant displacement of an atom during the entire course of an CMC19 run. Figure S3(d) indicates that a considerable number of atoms moved from their initial position at the beginning of the simulation to the final position at the end of the simulation via total atomic displacements as high as 10 Å. Since the resultant displacements associated with the accepted MC moves are mostly governed by the objective function in Eq. 1, it is unsurprising that the radial atomic correlation can develop up to a distance of the maximum displacement (i.e., $\approx$ 10 Å) of the atoms. This observation is indeed reflected in the radial distribution function (RDF), where the radial correlations between atomic pairs have been found to extend up to a distance of 10 Å or more (see Fig. S2).

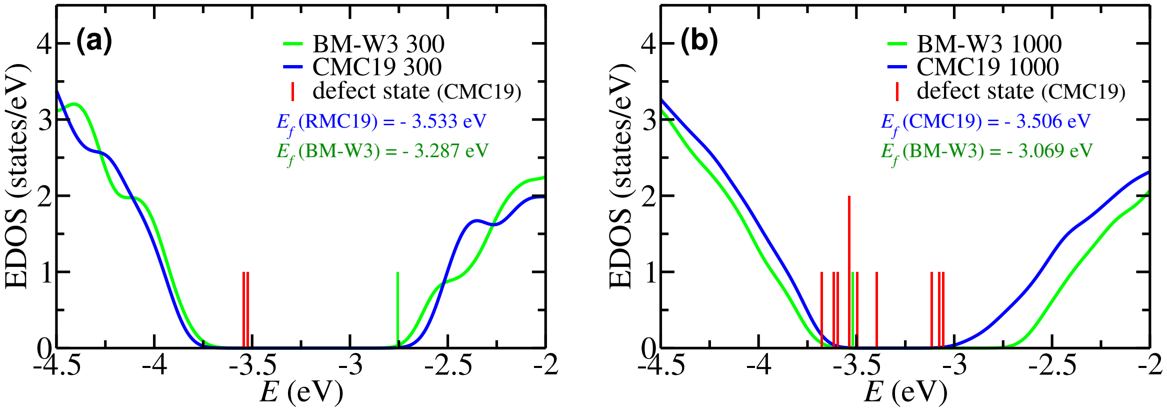

Figure S4. The EDOS of *a*-Si for: (a) 300-atom; and (b) 1000-atom CMC19 models (blue) and their corresponding BMW3 counterparts (green). The defect states from the CMC19 models are shown as red vertical lines, whereas the green vertical lines correspond to the edge states for the BMW3 models. The Fermi level is indicated as E_f in the plots.

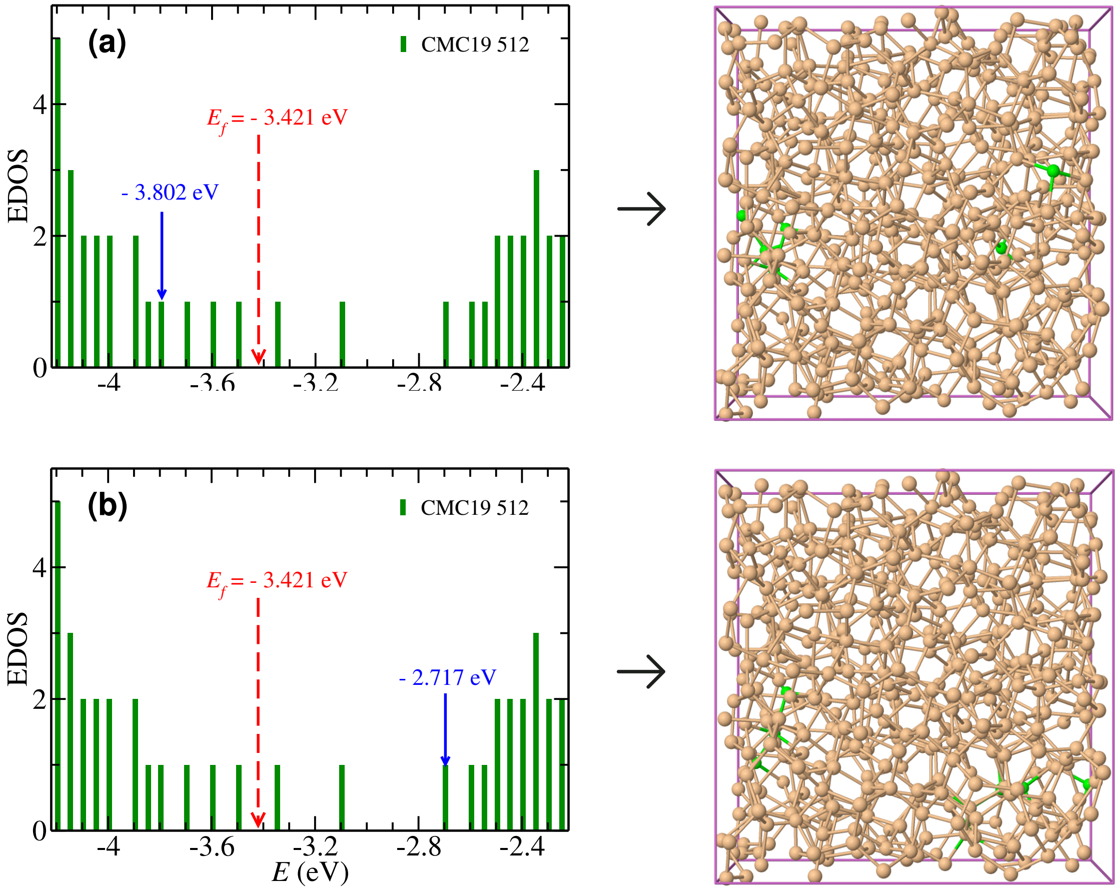

Figure S5. The electronic states near (a) the valence-band edge at -3.802 eV and (b) the conduction-band edge at -2.717 eV of a 512-atom CMC19 model. The atoms associated with these states are shown in green color. No dangling bonds are found to be associated with these edge states.

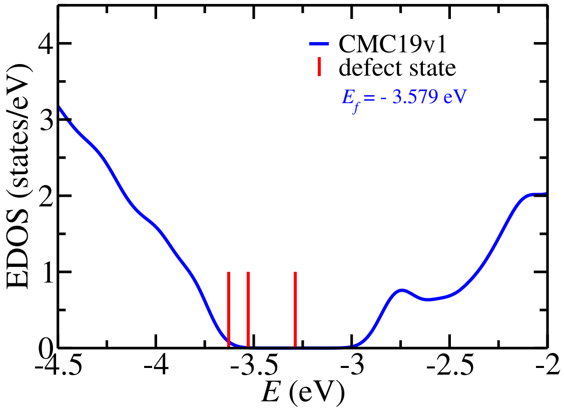

Figure S6. The EDOS of a 300-atom CMC19 model with a central void of radius 4 Å (see Fig. 4(b) in the main text). The defect states, shown as vertical lines (red), in the gap region originated from three dangling bonds present in the *ab initio*-relaxed model. The Fermi level is located at -3.579 eV.
